# (*Z*)-3-[(2-Amino­benz­yl)amino]-1-phenyl­but-2-en-1-one

**DOI:** 10.1107/S1600536814012070

**Published:** 2014-06-04

**Authors:** Vedavalli Sairaj, Thothadri Srinivasan, Muthusamy Kandaswamy, Devadasan Velmurugan

**Affiliations:** a Department of Inorganic Chemistry, University of Madras, Guindy Campus, Chennai 600 025, India; b Centre of Advanced Study in Crystallography and Biophysics, University of Madras, Guindy Campus, Chennai 600 025, India

## Abstract

In the title compound, C_17_H_18_N_2_O, the aromatic rings are almost normal to one another, making a dihedral angle of 89.00 (8)°. There is an intra­molecular N—H⋯O hydrogen bond in the mol­ecule enclosing an *S*(6) ring motif. In the crystal, mol­ecules are linked by N—H⋯O hydrogen bonds, forming chains along [010].

## Related literature   

For the biological activity of chalcones, see: Di Carlo *et al.* (1999 ▶); Lin *et al.* (2002 ▶). For a related structure, see: Ranjith *et al.* (2010 ▶).
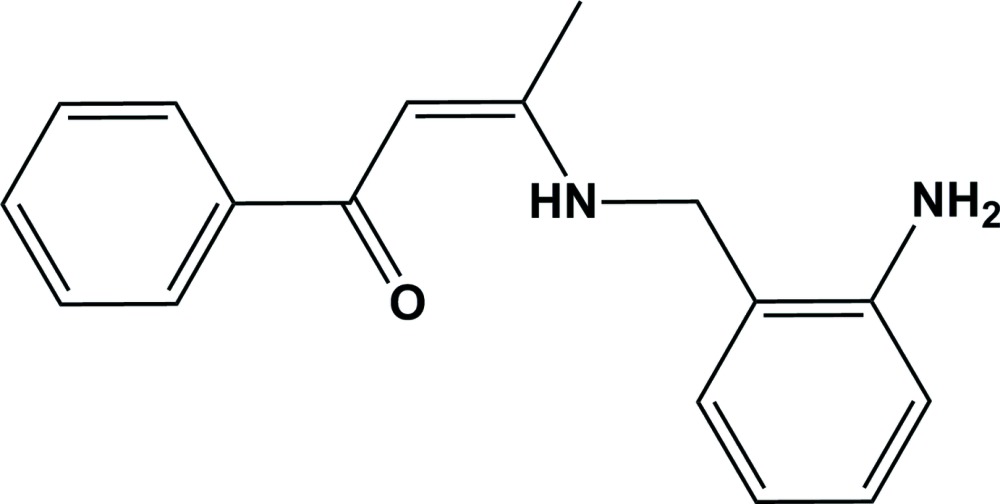


## Experimental   

### 

#### Crystal data  

C_17_H_18_N_2_O
*M* *_r_* = 266.33
Monoclinic, 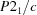

*a* = 11.3197 (4) Å
*b* = 9.8341 (3) Å
*c* = 13.4207 (4) Å
β = 106.387 (2)°
*V* = 1433.29 (8) Å^3^
*Z* = 4
Mo *K*α radiation
μ = 0.08 mm^−1^
*T* = 293 K
0.30 × 0.25 × 0.20 mm

#### Data collection  

Bruker SMART APEXII area-detector diffractometer
Absorption correction: multi-scan (*SADABS*; Bruker, 2008 ▶) *T*_min_ = 0.698, *T*_max_ = 0.746
14860 measured reflections
3921 independent reflections
2528 reflections with *I* > 2σ(*I*)
*R*_int_ = 0.028

#### Refinement  

*R*[*F* ^2^ > 2σ(*F* ^2^)] = 0.050
*wR*(*F* ^2^) = 0.156
*S* = 1.03
3921 reflections
183 parameters
H-atom parameters constrained
Δρ_max_ = 0.23 e Å^−3^
Δρ_min_ = −0.16 e Å^−3^

### 

Data collection: *APEX2* (Bruker, 2008 ▶); cell refinement: *SAINT* (Bruker, 2008 ▶); data reduction: *SAINT*; program(s) used to solve structure: *SHELXS97* (Sheldrick, 2008 ▶); program(s) used to refine structure: *SHELXL97* (Sheldrick, 2008 ▶); molecular graphics: *ORTEP-3 for Windows* (Farrugia, 2012 ▶); software used to prepare material for publication: *SHELXL97* and *PLATON* (Spek, 2009 ▶).

## Supplementary Material

Crystal structure: contains datablock(s) global, I. DOI: 10.1107/S1600536814012070/su2735sup1.cif

Structure factors: contains datablock(s) I. DOI: 10.1107/S1600536814012070/su2735Isup2.hkl

Supporting information file. DOI: 10.1107/S1600536814012070/su2735Isup3.cml

CCDC reference: 1005094

Additional supporting information:  crystallographic information; 3D view; checkCIF report

## Figures and Tables

**Table 1:** Hydrogen-bond geometry (Å, °)

*D*—H⋯*A*	*D*—H	H⋯*A*	*D*⋯*A*	*D*—H⋯*A*
N2—H2*A*⋯O1	0.86	1.99	2.6619 (17)	134
N1—H1*A*⋯O1^i^	0.86	2.27	3.0009 (19)	143

Symmetry code: (i) 
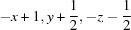
.

**Fig. 1.**
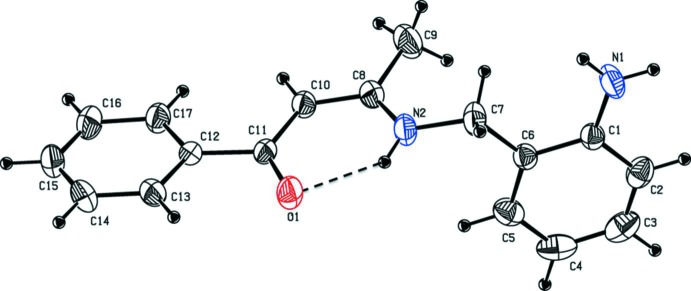
The molecular structure of the title compound, with atom labelling. Displacement ellipsoids are drawn at the 30% probability level. The intramolecular N-H···O hydrogen bond is shown as a dashed line (see Table 1 for details).

**Fig. 2.**
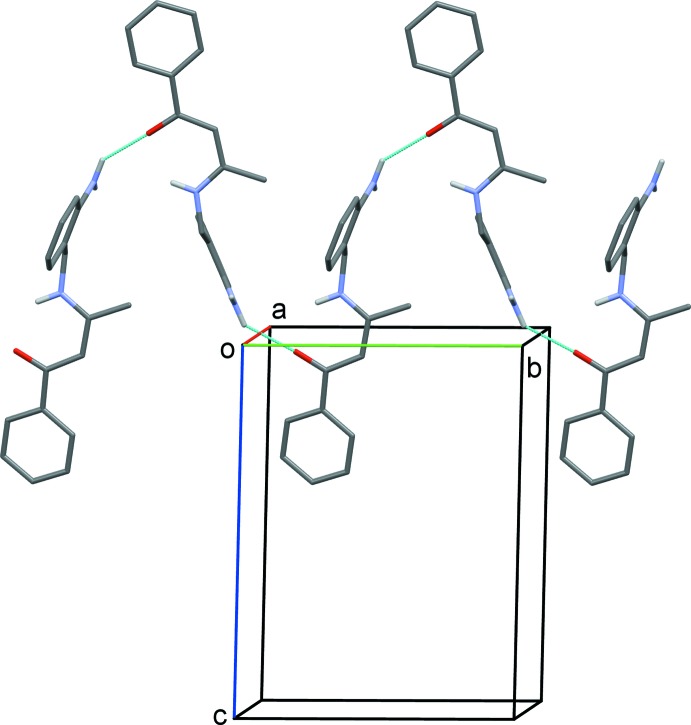
A partial view along the a axis of the crystal packing of the title compound. The hydrogen bonds are shown as dashed lines (see Table 1 for details).

## supplementary crystallographic information

### S1. Comment

Chalcones are a major class of natural products with widespread distribution in fruits, vegetables, spices, tea and soy based foodstuff and have recently been the subject of great interest for their interesting pharmacological activities (Di Carlo *et al.*, 1999). Chalcones and flavonoids have been reported to be active anti-tuberculosis agents (Lin *et al.*,2002). Against this background and in order to obtain detailed information on molecular conformation in the solid state, an X-ray study of the title compound was carried out.

In the title compound, Fig. 1, the aminobenzyl ring (C1-C6) and the phenyl ring (C12-C17) are normal to one another with a dihedral angle of 89.00 (8)°. The amine N atom, N1, attached to phenyl ring (C1-C6), deviates by only -0.0020 (16) Å from the ring plane. There is an intramolecular N-H···O hydrogen bonds enclosing an S(6) ring motif.

In the crystal, molecules are linked by N–H···O hydrogen bonds forming chains along the b axis direction (Table 1 and Fig. 2).

### S2. Experimental

To a warm ethanolic solution (25 ml) of 2-aminobenzylamine (0.25 g, 0.2 mmol), an ethanolic solution of benzylacetone (0.3 g, 0.2 mmol) was added dropwise and the resulting solution was refluxed for 3 h. The solution was then filtered hot and allowed to stand at room temperature. After slow evaporation of the solvent at 298 K, block-like colourless crystals of the title compound were obtained. They were filtered off, washed with cold methanol and dried [Yield 0.45 g, 83%].

### S3. Refinement

Hydrogen atoms were placed in calculated positions and refined as riding atoms: N-H = 0.86 Å, C—H = 0.93- 0.97 Å, with U*iso*(H) = 1.5U*eq*(C-methyl) and = 1.2U*eq*(N,C) for other H atoms.

### Crystal data

**Table:**

C_17_H_18_N_2_O	*F*(000) = 568
*M**_r_* = 266.33	*D*_x_ = 1.234 Mg m^−^^3^
Monoclinic, *P*2_1_/*c*	Mo *K*α radiation, λ = 0.71073 Å
Hall symbol: -P 2ybc	Cell parameters from 3921 reflections
*a* = 11.3197 (4) Å	θ = 1.9–29.3°
*b* = 9.8341 (3) Å	µ = 0.08 mm^−^^1^
*c* = 13.4207 (4) Å	*T* = 293 K
β = 106.387 (2)°	Block, colourless
*V* = 1433.29 (8) Å^3^	0.30 × 0.25 × 0.20 mm
*Z* = 4	

### Data collection

**Table:**

Bruker SMART APEXII area-detector diffractometer	3921 independent reflections
Radiation source: fine-focus sealed tube	2528 reflections with *I* > 2σ(*I*)
Graphite monochromator	*R*_int_ = 0.028
ω and φ scans	θ_max_ = 29.3°, θ_min_ = 1.9°
Absorption correction: multi-scan (*SADABS*; Bruker, 2008)	*h* = −15→15
*T*_min_ = 0.698, *T*_max_ = 0.746	*k* = −13→13
14860 measured reflections	*l* = −10→18

### Refinement

**Table:**

Refinement on *F*^2^	Secondary atom site location: difference Fourier map
Least-squares matrix: full	Hydrogen site location: inferred from neighbouring sites
*R*[*F*^2^ > 2σ(*F*^2^)] = 0.050	H-atom parameters constrained
*wR*(*F*^2^) = 0.156	*w* = 1/[σ^2^(*F*_o_^2^) + (0.0715*P*)^2^ + 0.2241*P*] where *P* = (*F*_o_^2^ + 2*F*_c_^2^)/3
*S* = 1.03	(Δ/σ)_max_ < 0.001
3921 reflections	Δρ_max_ = 0.23 e Å^−^^3^
183 parameters	Δρ_min_ = −0.16 e Å^−^^3^
0 restraints	Extinction correction: *SHELXL97* (Sheldrick, 2008), Fc^*^=kFc[1+0.001xFc^2^λ^3^/sin(2θ)]^-1/4^
Primary atom site location: structure-invariant direct methods	Extinction coefficient: 0.020 (3)

### Special details

**Table:**

Geometry. All esds (except the esd in the dihedral angle between two l.s. planes) are estimated using the full covariance matrix. The cell esds are taken into account individually in the estimation of esds in distances, angles and torsion angles; correlations between esds in cell parameters are only used when they are defined by crystal symmetry. An approximate (isotropic) treatment of cell esds is used for estimating esds involving l.s. planes.
Refinement. Refinement of F^2^ against ALL reflections. The weighted R-factor wR and goodness of fit S are based on F^2^, conventional R-factors R are based on F, with F set to zero for negative F^2^. The threshold expression of F^2^ > 2sigma(F^2^) is used only for calculating R-factors(gt) etc. and is not relevant to the choice of reflections for refinement. R-factors based on F^2^ are statistically about twice as large as those based on F, and R- factors based on ALL data will be even larger.

### Fractional atomic coordinates and isotropic or equivalent isotropic displacement parameters (Å^2^)

**Table:**

	*x*	*y*	*z*	*U*_iso_*/*U*_eq_	
C1	0.37143 (13)	0.36601 (14)	−0.34891 (10)	0.0447 (3)	
C2	0.24361 (15)	0.37189 (17)	−0.38045 (13)	0.0588 (4)	
H2	0.2035	0.4073	−0.4455	0.071*	
C3	0.17631 (16)	0.3268 (2)	−0.31782 (17)	0.0701 (5)	
H3	0.0908	0.3311	−0.3407	0.084*	
C4	0.23319 (19)	0.27475 (19)	−0.22096 (17)	0.0724 (5)	
H4	0.1867	0.2442	−0.1783	0.087*	
C5	0.36069 (17)	0.26853 (18)	−0.18788 (13)	0.0601 (4)	
H5	0.3995	0.2335	−0.1224	0.072*	
C6	0.43142 (13)	0.31337 (15)	−0.25025 (10)	0.0445 (3)	
C7	0.56975 (14)	0.3028 (2)	−0.21832 (11)	0.0602 (4)	
H7A	0.5930	0.2264	−0.2545	0.072*	
H7B	0.6031	0.3844	−0.2407	0.072*	
C8	0.66303 (14)	0.38414 (16)	−0.03882 (11)	0.0516 (4)	
C9	0.6376 (2)	0.5280 (2)	−0.07480 (15)	0.0830 (6)	
H9A	0.6843	0.5498	−0.1222	0.125*	
H9B	0.6607	0.5880	−0.0161	0.125*	
H9C	0.5513	0.5384	−0.1092	0.125*	
C10	0.72605 (14)	0.35770 (15)	0.06411 (11)	0.0492 (4)	
H10	0.7488	0.4313	0.1090	0.059*	
C11	0.75754 (13)	0.22683 (14)	0.10462 (10)	0.0438 (3)	
C12	0.83849 (12)	0.20920 (14)	0.21386 (10)	0.0414 (3)	
C13	0.88339 (15)	0.08133 (17)	0.24592 (12)	0.0559 (4)	
H13	0.8632	0.0086	0.1999	0.067*	
C14	0.95824 (18)	0.0598 (2)	0.34596 (13)	0.0714 (5)	
H14	0.9888	−0.0267	0.3663	0.086*	
C15	0.98725 (17)	0.1654 (2)	0.41484 (12)	0.0709 (5)	
H15	1.0376	0.1509	0.4819	0.085*	
C16	0.94187 (17)	0.2926 (2)	0.38466 (12)	0.0691 (5)	
H16	0.9604	0.3641	0.4318	0.083*	
C17	0.86870 (15)	0.31536 (17)	0.28458 (11)	0.0568 (4)	
H17	0.8395	0.4025	0.2645	0.068*	
N1	0.43740 (14)	0.41274 (17)	−0.41456 (10)	0.0704 (4)	
H1A	0.3993	0.4452	−0.4745	0.084*	
H1B	0.5165	0.4093	−0.3954	0.084*	
N2	0.62611 (13)	0.28532 (14)	−0.10721 (9)	0.0572 (4)	
H2A	0.6362	0.2033	−0.0841	0.069*	
O1	0.72160 (13)	0.12117 (11)	0.05299 (8)	0.0710 (4)	

### Atomic displacement parameters (Å^2^)

**Table:**

	*U*^11^	*U*^22^	*U*^33^	*U*^12^	*U*^13^	*U*^23^
C1	0.0496 (8)	0.0388 (8)	0.0424 (7)	0.0033 (6)	0.0076 (6)	−0.0008 (5)
C2	0.0501 (9)	0.0528 (9)	0.0654 (9)	0.0066 (7)	0.0031 (7)	−0.0081 (7)
C3	0.0493 (9)	0.0607 (11)	0.0986 (14)	−0.0023 (8)	0.0179 (9)	−0.0194 (10)
C4	0.0741 (12)	0.0607 (11)	0.0985 (14)	−0.0168 (9)	0.0506 (11)	−0.0133 (10)
C5	0.0728 (11)	0.0578 (10)	0.0551 (8)	−0.0043 (8)	0.0270 (8)	0.0019 (7)
C6	0.0493 (8)	0.0446 (8)	0.0381 (6)	0.0003 (6)	0.0101 (5)	0.0000 (6)
C7	0.0511 (8)	0.0879 (13)	0.0373 (7)	0.0072 (8)	0.0052 (6)	0.0062 (7)
C8	0.0517 (8)	0.0490 (9)	0.0492 (7)	0.0033 (7)	0.0065 (6)	0.0074 (6)
C9	0.1066 (16)	0.0575 (12)	0.0705 (11)	0.0052 (11)	0.0013 (11)	0.0193 (9)
C10	0.0554 (8)	0.0400 (8)	0.0446 (7)	−0.0008 (6)	0.0016 (6)	0.0002 (6)
C11	0.0445 (7)	0.0414 (8)	0.0402 (6)	−0.0044 (6)	0.0032 (5)	−0.0026 (5)
C12	0.0389 (7)	0.0425 (8)	0.0401 (6)	−0.0038 (5)	0.0069 (5)	0.0023 (5)
C13	0.0635 (10)	0.0474 (9)	0.0527 (8)	0.0000 (7)	0.0094 (7)	0.0081 (7)
C14	0.0772 (12)	0.0672 (12)	0.0623 (10)	0.0094 (9)	0.0074 (9)	0.0260 (9)
C15	0.0655 (11)	0.0922 (14)	0.0453 (8)	−0.0024 (10)	0.0001 (7)	0.0165 (9)
C16	0.0724 (11)	0.0783 (13)	0.0455 (8)	−0.0077 (10)	−0.0015 (8)	−0.0074 (8)
C17	0.0621 (9)	0.0502 (9)	0.0482 (8)	−0.0006 (7)	−0.0008 (7)	−0.0032 (7)
N1	0.0644 (9)	0.0951 (12)	0.0499 (7)	0.0127 (8)	0.0134 (6)	0.0302 (7)
N2	0.0638 (8)	0.0585 (8)	0.0395 (6)	0.0052 (6)	−0.0012 (6)	0.0044 (5)
O1	0.0959 (9)	0.0427 (7)	0.0538 (6)	−0.0044 (6)	−0.0122 (6)	−0.0065 (5)

### Geometric parameters (Å, º)

**Table:**

C1—N1	1.384 (2)	C9—H9C	0.9600
C1—C2	1.389 (2)	C10—C11	1.403 (2)
C1—C6	1.4053 (18)	C10—H10	0.9300
C2—C3	1.358 (3)	C11—O1	1.2516 (16)
C2—H2	0.9300	C11—C12	1.5036 (17)
C3—C4	1.376 (3)	C12—C13	1.379 (2)
C3—H3	0.9300	C12—C17	1.387 (2)
C4—C5	1.386 (3)	C13—C14	1.387 (2)
C4—H4	0.9300	C13—H13	0.9300
C5—C6	1.384 (2)	C14—C15	1.367 (3)
C5—H5	0.9300	C14—H14	0.9300
C6—C7	1.506 (2)	C15—C16	1.369 (3)
C7—N2	1.4571 (17)	C15—H15	0.9300
C7—H7A	0.9700	C16—C17	1.383 (2)
C7—H7B	0.9700	C16—H16	0.9300
C8—N2	1.3210 (19)	C17—H17	0.9300
C8—C10	1.3888 (19)	N1—H1A	0.8600
C8—C9	1.496 (2)	N1—H1B	0.8600
C9—H9A	0.9600	N2—H2A	0.8600
C9—H9B	0.9600		
			
N1—C1—C2	119.65 (13)	H9B—C9—H9C	109.5
N1—C1—C6	121.19 (13)	C8—C10—C11	124.07 (13)
C2—C1—C6	119.16 (14)	C8—C10—H10	118.0
C3—C2—C1	121.04 (16)	C11—C10—H10	118.0
C3—C2—H2	119.5	O1—C11—C10	122.65 (12)
C1—C2—H2	119.5	O1—C11—C12	117.23 (12)
C2—C3—C4	120.75 (16)	C10—C11—C12	120.12 (12)
C2—C3—H3	119.6	C13—C12—C17	118.34 (13)
C4—C3—H3	119.6	C13—C12—C11	118.62 (13)
C3—C4—C5	119.09 (16)	C17—C12—C11	123.03 (13)
C3—C4—H4	120.5	C12—C13—C14	120.79 (16)
C5—C4—H4	120.5	C12—C13—H13	119.6
C6—C5—C4	121.33 (16)	C14—C13—H13	119.6
C6—C5—H5	119.3	C15—C14—C13	120.23 (17)
C4—C5—H5	119.3	C15—C14—H14	119.9
C5—C6—C1	118.63 (14)	C13—C14—H14	119.9
C5—C6—C7	122.58 (13)	C14—C15—C16	119.68 (15)
C1—C6—C7	118.75 (12)	C14—C15—H15	120.2
N2—C7—C6	114.79 (13)	C16—C15—H15	120.2
N2—C7—H7A	108.6	C15—C16—C17	120.48 (16)
C6—C7—H7A	108.6	C15—C16—H16	119.8
N2—C7—H7B	108.6	C17—C16—H16	119.8
C6—C7—H7B	108.6	C16—C17—C12	120.47 (16)
H7A—C7—H7B	107.5	C16—C17—H17	119.8
N2—C8—C10	121.76 (14)	C12—C17—H17	119.8
N2—C8—C9	118.48 (13)	C1—N1—H1A	120.0
C10—C8—C9	119.75 (15)	C1—N1—H1B	120.0
C8—C9—H9A	109.5	H1A—N1—H1B	120.0
C8—C9—H9B	109.5	C8—N2—C7	125.86 (14)
H9A—C9—H9B	109.5	C8—N2—H2A	117.1
C8—C9—H9C	109.5	C7—N2—H2A	117.1
H9A—C9—H9C	109.5		
			
N1—C1—C2—C3	179.97 (16)	C8—C10—C11—C12	172.88 (14)
C6—C1—C2—C3	−0.5 (2)	O1—C11—C12—C13	9.5 (2)
C1—C2—C3—C4	0.4 (3)	C10—C11—C12—C13	−170.01 (14)
C2—C3—C4—C5	−0.2 (3)	O1—C11—C12—C17	−169.53 (15)
C3—C4—C5—C6	0.0 (3)	C10—C11—C12—C17	11.0 (2)
C4—C5—C6—C1	0.0 (2)	C17—C12—C13—C14	−0.9 (2)
C4—C5—C6—C7	−177.47 (16)	C11—C12—C13—C14	−179.89 (15)
N1—C1—C6—C5	179.81 (15)	C12—C13—C14—C15	0.9 (3)
C2—C1—C6—C5	0.3 (2)	C13—C14—C15—C16	0.1 (3)
N1—C1—C6—C7	−2.6 (2)	C14—C15—C16—C17	−1.1 (3)
C2—C1—C6—C7	177.83 (14)	C15—C16—C17—C12	1.1 (3)
C5—C6—C7—N2	−19.4 (2)	C13—C12—C17—C16	−0.1 (2)
C1—C6—C7—N2	163.10 (14)	C11—C12—C17—C16	178.86 (15)
N2—C8—C10—C11	0.8 (3)	C10—C8—N2—C7	−174.01 (15)
C9—C8—C10—C11	−178.05 (17)	C9—C8—N2—C7	4.9 (3)
C8—C10—C11—O1	−6.6 (3)	C6—C7—N2—C8	−90.1 (2)

### Hydrogen-bond geometry (Å, º)

**Table:**

*D*—H···*A*	*D*—H	H···*A*	*D*···*A*	*D*—H···*A*
N2—H2*A*···O1	0.86	1.99	2.6619 (17)	134
N1—H1*A*···O1^i^	0.86	2.27	3.0009 (19)	143

Symmetry code: (i) −*x*+1, *y*+1/2, −*z*−1/2.
